# Exploring the multifunctionality of SR proteins

**DOI:** 10.1042/BST20210325

**Published:** 2021-12-23

**Authors:** Irena Slišković, Hannah Eich, Michaela Müller-McNicoll

**Affiliations:** Institute for Molecular Bio Science, Goethe University Frankfurt, Max-von-Laue-Str. 13, 60438 Frankfurt/Main, Germany

**Keywords:** challenges, gene expression, multifunctionality, SR proteins

## Abstract

Members of the arginine–serine-rich protein family (SR proteins) are multifunctional RNA-binding proteins that have emerged as key determinants for mRNP formation, identity and fate. They bind to pre-mRNAs early during transcription in the nucleus and accompany bound transcripts until they are translated or degraded in the cytoplasm. SR proteins are mostly known for their essential roles in constitutive splicing and as regulators of alternative splicing. However, many additional activities of individual SR proteins, beyond splicing, have been reported in recent years. We will summarize the different functions of SR proteins and discuss how multifunctionality can be achieved. We will also highlight the difficulties of studying highly versatile SR proteins and propose approaches to disentangle their activities, which is transferrable to other multifunctional RBPs.

## Introduction

Serine/arginine-rich splicing factors (SRSFs, SR proteins) are a phylogenetically conserved family of RNA-binding proteins (RBPs) present in all metazoans and plants [1]. Initially described as essential regulators of constitutive and alternative pre-mRNA splicing [2,3], we now know that SR proteins influence all steps in the life cycle of mRNAs from transcription, splicing, polyadenylation and mRNP packing in the nucleus to mRNA export, translation and decay in the cytoplasm [3–5]. Apart from canonical and non-canonical functions in mRNA metabolism, SR proteins also participate in the processing of non-coding RNAs, the regulation of post-translational modifications (PTMs), and the formation and dynamics of nuclear compartments (see below) [6–9].

SR proteins are essential for normal development as gene knockouts (KOs) cause embryonic lethality in mice and *Drosophila* [3]. They maintain pluripotency of embryonic stem cells and show highest expression levels in undifferentiated cells [10]. SR protein levels are down-regulated during differentiation but their aberrant overexpression promotes dedifferentiation, tumorigenesis and metastasis [2]. Individual SR proteins are implicated in different cancers [2]; for example, SRSF1 is associated with acute lymphoblastic leukemia (ALL), prostate, lung and breast cancer [2,3], SRSF2 and SRSF4 with acute myeloid leukemia (AML) [2,11,12], SRSF3 with colon cancer and osteosarcoma [5,13], SRSF5 with lung and breast cancer [2,14] and SRSF6 with breast and skin cancer [2,14]. Dysregulation of canonical and non-canonical functions of SR proteins also gives rise to neurological disorders, liver disease as well as coronary and cardiac diseases [5,15–17].

Being involved in a plethora of different functions, SR proteins are truly all-rounders —multifunctional and highly versatile RBPs that connect and control subsequent steps in eukaryotic gene expression. Here, we will describe how this multifunctionality is achieved and what we need to consider to disentangle specific functions of individual SR proteins.

## The SR protein family

**Figure 1.**
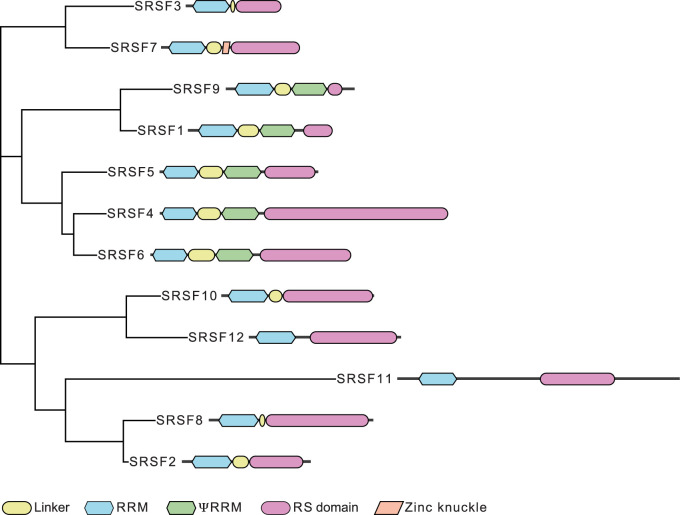
Phylogenetic tree of the SR protein family and their domain structure. Human SR protein sequences were obtained from UniProt (last modified Feb 2, 2021) [19] and aligned with mafft, v7, L-INS-I method [20,21]. A maximum likelihood phylogenetic tree was produced with RAxML, v1.0.0, using LG + G4 as substitution model [22]. The domains were produced with DoMosaics utilizing the hmmer and Pfam protein domain database [23–25]. The annotation*s* were manually curated according to [18].

The 12 canonical SR proteins contain at least one RNA recognition motif (RRM) at the N-terminus, a glycine-rich spacer region and a domain rich in arginines and serines (RS domain) at the C-terminus [3,18] (Figure 1). The RNA-binding specificity of SR proteins differs among the family members but is surprisingly promiscuous [6,18] (Table 1). It is determined by the number and spacing of the RRMs, their accessibility and the relative contribution of linker regions and additional domains.

**Table 1.** Consensus binding motifs and cellular functions of individual SR proteins

SR protein	Aliases	Binding motifs	Functions	References
SRSF1	ASF, SF2, SRp30a, SFRS1	RGAAGAAC AGGAC[A/G][G/A]AGC GAAGAA **GGAGGA**	Constitutive and alternative splicing activator, Transcriptional elongation, Genome stability, mRNA stability, nonsense-mediated decay, mRNA export, Translation, miRNA processing, Sumoylation, Nuclear speckle assembly	[6,18,26–35]
SRSF2	SC35, SRp30b, SFRS2	**[C/G][C/G]NG** AGGAGAU GUUCGAGUA UGCNG[C/U]	Constitutive and alternative splicing activator, Genome stability, nonsense-mediated decay, Transcriptional elongation	[3,6,30,31,36]
SRSF3	SRp20, SFRS3	[A/U]C[A/U][A/U]C CUC[U/G]UC[C/U] CA-rich **CNYC**	Constitutive and alternative splicing activator, Transcriptional termination, Nuclear decay, Alternative polyadenylation, mRNA export, mRNA translation, Assembly of stress granules and P-bodies, miRNA processing, nonsense-mediated decay	[3,6,18,27,30, 31,36–44]
SRSF4	SRp75, SFRS4	GAAGGA **GAAGAA**	Constitutive and alternative splicing activator, Transcription, inhibition of polyadenylation, ncRNA processing, nonsense-mediated decay	[6,30,31,43,45]
SRSF5	SRp40, HRS, SFRS5	GAGCAGUCGGCUC AC[A/C/U]G[G/C] **CUG**	Constitutive and alternative splicing activator, (viral) mRNA translation, mRNA export, nonsense-mediated decay	[6,30,31,36,46,47]
SRSF6	SRp55, B52, SFRS6	U[C/G]CG[U/G][A/C] UCAACCAGGCGAC **GAAGAA**	Constitutive and alternative splicing activator, (viral) mRNA translation, nonsense-mediated decay	[6,30,31,36,46,48]
SRSF7	9G8, SFRS7	UCAACA ACGAGAGA[C/U] GGACGACGAG **GAYGAY**	Constitutive and alternative splicing activator, mRNA export, alternative polyadenylation, (viral) mRNA processing and translation, miRNA processing, nuclear body assembly, nonsense-mediated decay	[6,30,31,36,37, 44,49–51]
SRSF8	SRp46, SFRS2B	ND	Constitutive and alternative splicing activator	[36,52]
SRSF9	SRp30c, SFRS9	GACGAC AAAGAGCUCGG CUGGAUU **GGAGGA**	Constitutive and alternative splicing regulator, mRNA translation, SUMOylation	[6,7,30,32,34,53]
SRSF10	SRp38, SRrp40, TASR1, SFRS13A	AAAGACAAA [A/T/G]GA[A/G][A/G][A/G]	Inducible, global splicing repressor mRNA translation	[6,18,36,54]
SRSF11	SRp54, p54, SFRS11	AAGAAG	Alternative splicing repressor, Genome stability	[6,30,36]
SRSF12	SRrp35, SFRS13B	ND	Positive and negative regulator of alternative splicing	[18,30]

The RS domain is composed of at least 50 amino acids with >40% RS dipeptide content and is intrinsically unstructured [55]. This domain mediates mainly protein–protein interactions with other RS domain-containing proteins but was also shown to interact with RNA [6]. The reversible phosphorylation of most serines within the RS domain is crucial for the regulation of SR protein activities. Fully phosphorylated RS domains are required for the recruitment of SR proteins to transcription sites and spliceosome assembly, while RS dephosphorylation promotes splicing catalysis, mRNP packaging and nuclear export [18,36]. Serving as a nuclear localization signal, the RS domain also regulates the subcellular localization and the nucleo-cytoplasmic shuttling of SR proteins [18].

## SR proteins and pre-mRNA splicing

Pre-mRNA splicing refers to the removal of introns and ligation of exons. Moreover, the differential inclusion of exons or introns can give rise to several alternative splice isoforms from one single gene. The major spliceosome, a multiprotein–RNA complex assembled from five small nuclear ribonucleoprotein particles (snRNPs, U1, U2, U4, U5 and U6), catalyzes the splicing reaction. SR proteins bind to splicing enhancers located in exons and introns and promote the recruitment of U1 snRNP to 5′ splice sites and U2 auxiliary factor (U2AF) to 3′ splice sites. Recruitment occurs in a phosphorylation-dependent manner through interactions with the U1 subunit U1-70k and U2AF65 via their RS domains. SR protein binding to intronic splicing silencers inhibits U1 and U2 recruitment. SR proteins also stabilize the base-pairing of U2 snRNP with the branchpoint and promote the recruitment of the U4/U6–U5 tri-snRNP and U6 snRNP binding. The catalytically active spliceosome is formed through extensive remodeling of RNA–RNA and RNA–protein interactions, which is coupled to the dephosphorylation of SR proteins [1,3,6,18,36,56].

Pre-mRNA splicing occurs co-transcriptionally, and SR proteins are stored in nuclear speckles until transcription is activated [56]. Release of SR proteins from nuclear speckles is regulated by phosphorylation of their RS domain. Hyper-phosphorylated SR proteins leave nuclear speckles, move to sites of active RNA polymerase II (Pol II) transcription, bind to pre-mRNAs and engage in co-transcriptional splicing [18]. SR proteins usually promote splice site usage depending on their binding strength, expression levels, and extent of cooperation and competition with other SR proteins and hnRNP proteins [36]. However, SR proteins sometimes repress splicing by binding to splicing silencers. SRSF10, SRSF11 and SRSF12 act as general splicing repressors when they become dephosphorylated under certain stresses [18,36,54].

## Non-canonical functions of SR proteins in the mRNA life cycle

**Figure 2.**
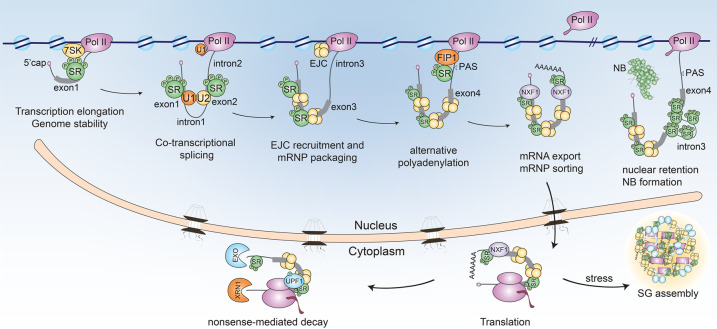
Canonical and non-canonical functions of SR proteins in the mRNA life cycle. Pol II, RNA polymerase II; EJC, exon junction complex; PAS, polyadenylation site; NB, nuclear body; SG, stress granule; EXO, exosome.

SR proteins perform diverse non-canonical functions that can be either related or unrelated to splicing [3]. While all SR proteins regulate pre-mRNA splicing in a partially redundant manner [6,30,36], non-canonical functions are often specific for a subset of SR proteins. The well-studied SR proteins SRSF1, SRSF2, SRSF3 and SRSF7 appear particularly versatile, each conveying a plethora of functions (Table 1, Figure 2). Other less-studied SR proteins may hide interesting novel functions.

During transcription, SR proteins interact with Pol II via its C-terminal domain (CTD) and contact nascent pre-mRNAs as soon as splice sites emerge [18]. SRSF2 is also part of the inhibitory 7SK snRNP complex that causes Pol II to pause at promoters and regulates transcription elongation. The emergence of SRSF2-binding sites in nascent pre-mRNAs causes SRSF2 to leave the 7SK complex, which destabilizes it and releases Pol II from pausing [3]. SRSF1 also regulates transcription, e.g. of HIV-1 genomic RNA during early stages of infection [35]. It also prevents the hybridization of newly synthesized pre-mRNA to the complementary DNA template during transcription (R loops), which reduces the occurrence of DNA double-strand breaks and maintains genome stability [28].

After completion of co-transcriptional splicing, dephosphorylated SR proteins stay bound to mRNAs and regulate downstream steps of gene expression [18]. They mark mRNAs as mature and export competent and assist in packaging and compaction of mRNPs through interactions with the exon-junction complex (EJC), which binds ∼20–24 nucleotides upstream of each exon–exon junction [57]. SRSF3 and SRSF7 modulate the length of 3′UTRs through alternative polyadenylation (APA) independently of splicing and enhance the RNA-binding capacity of the nuclear export factor NXF1 [31,44]. Several other SR proteins also recruit NXF1 via their linker domains and promote the nuclear export of bound target transcripts [18,31,47,58]. Differences in the shuttling capacities of SR proteins are due to the length and phosphorylation state of their RS domains and the ability to recruit NXF1 [18,47].

Once in the cytoplasm, SRSF1 activates translation of a subset of bound transcripts by recruiting the protein kinase mammalian target of rapamycin (mTOR) [18,32]. In contrast, SRSF3 represses translation of target RNAs, e.g. tumor suppressor PDCD4 [6,38]. SRSF9 also enhances the translation of β-catenin in an mTOR-dependent manner [6,32]. SRSF5 and SRSF6 stimulate the translation of gag proteins from unspliced HIV-1 mRNA, while SRSF3 and SRSF7 promote the translation of unspliced viral RNAs containing internal ribosomal entry sites (IRES) or a constitutive transport element (CTE) [36,51].

SR proteins also affect the stability of mRNAs in the cytoplasm by promoting or inhibiting nonsense-mediated decay (NMD). This occurs either directly by promoting the deposition of EJCs downstream of premature termination codons (PTCs) and by recruiting the NMD factor UPF1, or indirectly through unproductive splicing and selective export [18,29]. Unproductive splicing occurs when SR proteins promote the removal of introns located within the 3′ UTRs of transcripts or the inclusion of alternative exons containing a PTC, which both render transcripts sensitive to NMD [59].

## SR proteins and non-coding RNAs

SR proteins also interact with non-coding RNAs, such as small nucleolar RNAs (snoRNAs), microRNA (miRNAs) and long non-coding RNAs (lncRNAs) [43,60]. Binding of SRSF1 and SRSF3 promotes miRNA processing by enhancing Drosha-mediated cleavage in a splicing-independent manner [8,27,33,39]. SRSF1 regulates maturation of miR-7, which targets the 3′UTR of *SRSF1* and represses its own translation [61]. It also influences the conformation of an inhibitory stem loop in the pri-miR-10b, which enables the hairpin selection by the Microprocessor [33]. SRSF3 binds to a specific pri-miRNA binding motif (CNNC) [49], and enhances the processing of CNNC-containing pri-miRNAs through Drosha recruitment [40].

By interacting with lncRNAs, SR proteins also contribute to the formation and dynamics of nuclear compartments. For example, the lncRNA metastasis-associated lung adenocarcinoma transcript 1 (*MALAT1*) localizes to nuclear speckles where it interacts with all SR proteins [3]. SRSF1 and *MALAT1* assemble nuclear speckles, and *MALAT1* regulates the levels and phosphorylation of SRSF1 [34]. However, different SR proteins have distinct binding sites and show distinct effects on *MALAT1* and its nuclear speckle localization and functions [62]. SRSF1 and SRSF9 assemble nuclear stress bodies (nSBs) when cells are exposed to high temperatures [7]. Heat stress induces transcription of non-coding *HSATIII* transcripts and SR protein dephosphorylation [63]. Dephosphorylated SRSF1 and SRSF9 bind to *HSATIII* lncRNAs, which consist mainly of GGAAU repeats and are sequestered in nSBs [7]. Recruitment of the SR protein-specific kinase CLK1 to nSBs upon stress relief enhances the selective re-phosphorylation of SRSF1 and SRSF9 and promotes their release from nSBs to regulate splicing of heat-shock response genes [7]. SRSF7 also assembles nuclear bodies as part of an intricate auto-regulatory feedback mechanism [50]. Overexpression of SRSF7 promotes the inclusion of an alternative exon containing a PTC and a downstream (re)initiation codon resulting in a bi-cistronic *SRSF7* transcript that is NMD resistant. Translation yields two SRSF7 protein halves precisely separating the RNA-binding domains from the RS domain (Split-ORFs). Accumulation of the first SRSF7 half in the nucleus inhibits splicing and *SRSF7* Split-ORF production and instead causes the retention of a specific intron containing 76 SRSF7 binding motifs. Repeated binding of SRSF7 to this intron and oligomerization of bound SRSF7 leads to the assembly of large nuclear bodies which sequester SRSF7 proteins and *SRSF7* transcripts and restores SRSF7 homeostasis [50]. It remains to be tested whether other SR proteins with a similar gene architecture also assemble nuclear bodies.

Some SR proteins (SRSF1, SRSF2, SRSF3, SRSF7 and SRSF10) are recruited to cytoplasmic stress granules (SGs) along with non-translated mRNAs [26]. For SRSF1, recruitment occurs through binding to its target transcripts [26], whereas SRSF3 appears to regulate SG and P-body assembly [5]. Neddylation of specific lysines in SRSF3 is crucial for the assembly of SGs upon arsenite stress [41]. Disassembly of SGs upon stress relief requires the small ubiquitin-related modifier (SUMO) pathway [64,65]. SRSF1 promotes SUMOylation either by recruiting the SUMO conjugating enzyme UBC9 or by regulating the activity of the SUMO E3 ligase PIAS1 [26,66–68]. SRSF1 also influences the SUMOylation of specific RBPs and spliceosomal components, which relies on its RRM2 domain [9,26].

The functional flexibility of the SR protein family is truly impressive and crucial for the plasticity, robustness and adaptability of gene expression programs. In the following chapters, we will highlight how SR proteins are able to perform their unique functions and achieve multifunctionality.

## RS domains as versatile protein-interaction hubs

The RS domain is a highly versatile and tunable platform for protein–protein interactions and one of the main reasons for the multifunctionality of SR proteins. Differential phosphorylation regulates protein interactions, RNA-binding specificity and nucleo-cytoplasmic shuttling of SR proteins [18]. RS domains interact preferentially with other RS or RS-like domains (RE/RD) that are found in most splicing factors [69]. However, several non-splicing factors, including the cleavage and polyadenylation (CPA) factors FIP1 and CPSF6, the m^6^A reader YTHDC1 and the the metallo-oxygenase JMJD6, also contain RS-like domains. They are known interactors of specific SR proteins [44,70–72] and enable their non-splicing functions. RS domains allow promiscuous but also specific interactions through differences in number, length and spacing of RS dipeptides, interspersed hydrophobic residues and the presence of specific domains embedded within the RS domain.

## Unique protein features

Additional functions are also possible when individual SR proteins contain unique protein domains or sequence features that increase their RNA-binding specificity, enable unique protein interactions or mediate distinct subcellular localizations [18]. For example, a zinc knuckle is found exclusively in SRSF7, which changes its RNA-binding specificity from CNYC to GAY triplets [44] and allows modulation of its binding to RNA by iron levels [73]. An additional pseudo RRM (ΨRRM), present in SRSF1, SRSF4, SRSF5, SRSF6 and SRSF9 (Figure 1), was shown to determine the RNA-binding specificity and splicing activity of SRSF1 [6,74]. The length and amino acid composition of the linker regions and RS domains also differ between SR proteins [18]. The unique RS domain of SRSF1 regulates the subnuclear localization of topoisomerase I, which was not observed for other SR proteins [75]. A unique hydrophobic stretch of 27 amino acids in the RS domain of SRSF7 contributes to its ability to assemble NBs and regulate APA [44,50]. Other unique domains are the PAP-inhibitory domain found exclusively in SRSF4, which inhibits the activity of the poly(A) polymerase PAP *in vitro* [45], and the phosphatase resistance domain found exclusively in SRSF2, which prevents its dephosphorylation during splicing and inhibits its nucleo-cytoplasmic shuttling [60,76,77]. Interestingly, SRSF2 shuttles in undifferentiated cells, suggesting that the functionalities of unique SR protein domains depend on the cell type or state [47].

Recent Gencode annotations (v38, Ensembl 104) reveal a multitude of novel alternative isoforms for some canonical SR protein genes, including *SRSF5*, *SRSF6*, *SRSF10* and *SRSF11*. These isoforms have shorter or missing RS domains and unique N-termini, which could provide them with novel functionalities that await investigation.

## Unique and overlapping RNA-binding specificities

SR proteins come in evolutionarily related pairs with similar RNA-binding specificities and functions, which are distinct from those of other pairs (Figure 1) [1]. For example, SRSF1 and SRSF9 are structurally very similar and both bind to GGA triplets [74,78,79]. SRSF9 and SRSF1 regulate the same splicing events, bind strongly to repetitive sequences within *HSATIII* lncRNAs using their ΨRRM, and promote SUMOylation [7,9,32]. SRSF4 and SRSF6 are closely related and both bind to GAA triplets and can partially compensate for each other [31,48,80]. The RNA-binding specificities of the paralogs SRSF3 and SRSF7 are different (CNYC and GAY), and both proteins often act in an antagonistic manner. However, genome-wide identification of SRSF7 binding motifs suggest that SRSF7 can bind to both GAY and CNYC sequences [50]. This suggests that the contribution of the zinc knuckle to RNA binding varies and that both proteins could in principle compete for binding to CNYC motifs.

SRSF2 and its paralog SRSF8 likely display the most promiscuous RNA-binding specificity of all SR proteins (SSNG) [81]. Interestingly, a mutation in *SRSF2* (SRSF2^P95H^) associated with myelodysplastic syndromes skews the binding specificity of SRSF2 from GGNG towards CCNG, affecting its binding to exons but not the global alternative splicing pattern [82]. This suggests that the main function of SRSF2 is probably to enhance the transcription of pre-mRNAs, which requires a promiscuous RNA-binding specificity, rather than regulating alternative splicing [3]. Accordingly, the lack of SRSF2-binding sites in most lncRNAs could contribute to their low abundance [83].

## mRNA modifications

SR protein functions are also modulated by N6-methyladenosine (m^6^A), the most abundant internal modification of mRNAs [2]. The methylation of adenosines in the vicinity of SR protein-binding sites could prevent or enhance SR protein binding and thus overwrite the expected outcome of gene regulation. m^6^A-mediated weakening of secondary structures may expose single-stranded RNA regions where SR proteins could bind [84], or m^6^A-bound reader proteins could recruit or repel SR proteins. For example, the nuclear m^6^A reader YTHDC1 selectively recruits dephosphorylated SRSF3 to neighboring splice sites and simultaneously blocks binding of SRSF10, thereby promoting the inclusion of specific exons [70,85]. The GGAAU repeats of *HSATIII* lncRNAs are partially m^6^A modified, and while unmodified GGAAU preferentially binds SRSF9, m^6^A-modified repeats are bound by YTHDC1, assembling distinct nSBs [7]. YTHDC1 also directs m^6^A-modified transcripts towards faster nuclear export through its interaction with SRSF3 and NXF1 recruitment [86]. Thus, through mutually exclusive interactions with specific SR proteins, YTHDC1 confers an additional layer of regulation and might modulate other non-canonical functions of SR proteins, such as APA, translation, nuclear body dynamics and mRNA decay.

## Binding to intronless transcripts and untranslated regions

SR proteins bind preferentially to exons, in a distance of 30–60 nt from splice sites [31,43], which allows their dephosphorylation during splicing. When SR proteins bind to intronless transcripts or far away from splice sites, e.g. within 5′ or 3′ untranslated regions (UTRs), they likely perform splicing-independent functions. For example, fully phosphorylated SRSF3 inhibits the translation of LPS-induced transcripts in microglia when it binds within their 3′UTRs [87]. SRSF1 binding within 3′UTRs of inflammation-related mRNAs prevents their nuclear export in murine macrophages [88]. Similarly, the insertion of purine-rich (GAA) SR protein-binding sites into intronless transcripts causes their retention in the nucleus [89]. In fact, hyper-phosphorylated SR proteins interact preferentially with nuclear retention factors, such as U1 snRNP, and fail to recruit NXF1 [20,47,90]. Surprisingly, sequence-specific binding of SRSF1/SRSF7 (GA-rich) to the intronless lncRNA *NKILA* and SRSF3/SRSF7 (CU-rich) to a 22-nt element in the coding region of intronless histone H2A mRNAs promotes their nuclear export through NXF1 recruitment [37,91].

SRSF3 and SRSF7 also regulate the length of 3′UTRs by binding upstream of proximal poly(A) sites (pPAS) and activating or inhibiting them in a splicing-independent manner [44]. SRSF7 is dephosphorylated during CPA, likely by phosphatases that are associated with the CPA complex [92], and subsequent recruitment of NXF1 could sort transcripts with different 3′UTR lengths prior to nuclear export [31]. In the absence of dedicated export factors, SRSF3 was shown to promote the degradation of intronless viral transcripts via the nuclear exosome [42], but it is unknown whether this is also true for cellular mRNAs.

## Post-translational modifications

Unique PTMs also contribute to the multifunctionality of individual SR proteins. PTMs are reversible and dynamically regulated under stress and they can alter RNA-binding specificity, protein interactors, protein stability and subcellular localization of SR proteins. In addition to serine phosphorylation within the RS domain, lysine acetylation, arginine methylation, neddylation, proline hydroxylation, ubiquitination and SUMOylation influence SR protein functions [9,18]. For example, Tip60-mediated acetylation of lysine 52 within the RRM of SRSF2 decreases its levels through enhanced proteasomal degradation [18,93]. In contrast, Tip60-mediated acetylation of lysine 125 within the ΨRRM of SRSF5 protects it from proteasomal degradation by antagonizing Smurf1-mediated ubiquitylation of the same residue [94]. SRSF2 protein stability is also regulated through the hydroxylation of proline residues within its RRM [95]. Arginine methylation within the linker regions of SRSF1 and SRSF5 influences their subcellular localization and shuttling capacity [47], while arginine methylation within the RRM enhances the RNA-binding capacity of SRSF2 [96]. Neddylation (Nedd8) was detected on SRSF1 and SRSF3, and neddylation of lysine 85 in the linker region of SRSF3 was shown to be crucial for the assembly of SGs [41].

## Challenges and opportunities in studying multifunctional SR proteins

Classical SR proteins are structurally very similar, and the functions of paralogs are especially difficult to tease apart. Furthermore, many SR-like proteins play roles in splicing, chromatin remodeling, and transcription [30]. A dense network of auto- and cross-regulation among SR- and SR-like proteins has recently been discovered, where the removal of one SR protein affects the levels of several others [10,43,97]. SR proteins interact with each other, co-operate and compete for binding sites and compensate for the loss or increased abundance of other SR proteins [78]. More distantly related SR proteins tend to co-operate, act in pairs or positively regulate each other's expression, while closely related SR proteins tend to compete or negatively regulate each other [10,31,44]. To discriminate between the functions of individual SR protein paralogs, we suggest endogenous tagging, e.g. with GFP. To minimize compensation and cross-regulation, acute degradation of individual SR proteins, e.g. through auxin-inducible degrons, should be used [98]. Short depletion times should help to dissect indirect from direct effects.

The activities of SR proteins depend on the phosphorylation of their RS domains and their subcellular localization, which rapidly changes in response to stress and signaling [18,99]. This should be considered in all functional studies. However, phosphoproteomics with SR proteins are particularly challenging due to their high number of potential phosphosites, the repetitive nature of their RS domain, their high charge (which impairs mobility and detection) and high arginine content (which precludes most peptide digestion strategies). However, individual SR proteins could be purified from mammalian cells and analyzed by native mass spectrometry (MS), which allows analysis of intact SR proteins and visualization of the distribution and number of phosphosites in the mass spectra [100].

It remains particularly challenging to disentangle the specific contributions of individual SR protein functions to gene expression and disease. Reduced levels of a target protein result from all gene expression steps that SR proteins regulate or connect, and loss-of-function or overexpression approaches will affect all of them. Thus, experiments must be designed very carefully to tease their different functions apart. Domain-swap or mutation experiments that inhibit interconnected pathways in *cis* may be useful. For example, Haward et al. [101] created a mouse model where they fused the phosphatase resistance domain of SRSF2 to SRSF1, thereby converting SRSF1 into a non-shuttling protein while preserving all its nuclear functions and allowing them to determine the contribution of SRSF1 shuttling on gene expression. Interconnected pathways can also be inhibited globally. For example, inhibition of the NMD pathway allowed to examine the contribution of all SR protein isoforms (including unstable ones) to alternative splicing [102], whereas inhibition of the mRNA export pathway allowed to assess the roles of SRSF3 in nuclear mRNA degradation of intronless viral transcripts [42]. Another possibility to separate non-canonical functions of SR proteins from their roles in splicing is the use of intronless reporter genes. With this approach, we have shown that 3'UTR APA regulation by SRSF7 is splicing independent [44]. Artificial SR proteins would also be a very interesting option to study the functions of individual SR proteins at specific splice sites or in UTRs by recruiting their RS domain fused to catalytically inactive dCasRX using specific guide RNAs [10,103].

## Perspectives

- SR proteins are essential multifunctional RBPs that have emerged as molecular adapters connecting and controlling gene expression and processing machineries at each step of the mRNA life cycle.
- Non-splicing functions are described for only a few well-studied members of the SR protein family and their individual contribution to total gene expression remains largely unknown.
- To better understand the contribution of individual SR proteins and their diverse roles in human disease, we need to develop methods and approaches to dissect the post-transcriptional and post-translational networks of SR proteins, study their functions at the single-cell level, explore their regulation by lncRNAs and subcellular compartments (riboregulation) and disentangle their non-splicing from their splicing functions. Also, better characterizing the less-studied family members and alternative splice isoforms will help us discover novel cellular functions and mechanisms.

## Abbreviations

ALL: acute lymphoblastic leukemia
CPA: cleavage and polyadenylation
EJC: exon-junction complex
lncRNAs: long non-coding RNAs
miRNAs: microRNA
mTOR: mammalian target of rapamycin
NMD: nonsense-mediated decay
nSBs: nuclear stress bodies
PTCs: premature termination codons
PTMs: post-translational modifications
RBPs: RNA-binding proteins
RRM: RNA recognition motif
SGs: stress granules
snRNPs: small nuclear ribonucleoprotein particles
SUMO: small ubiquitin-related modifier
UTRs: untranslated regions
